# Relationship Between Loneliness and Depression Among Chinese Junior High School Students: The Serial Mediating Roles of Internet Gaming Disorder, Social Network Use, and Generalized Pathological Internet Use

**DOI:** 10.3389/fpsyg.2020.529665

**Published:** 2021-02-12

**Authors:** Peng Wang, Jun Wang, Yun Yan, Yingdong Si, Xiangping Zhan, Yu Tian

**Affiliations:** Department of Psychology, Shandong Normal University, Jinan, China

**Keywords:** loneliness, internet gaming disorder, social network use, depression, multiple mediating effects, generalized pathological internet use

## Abstract

This study aimed to explore the mediating effects of internet gaming disorder, social network use, and generalized pathological internet use (GPIU) on the association between loneliness and depression. A total of 2211 junior high school students completed questionnaires regarding loneliness, internet gaming disorder, social network use, GPIU, and depression (aged 10–16 years). The results of a structural equation model revealed that (a) the path coefficient of loneliness to depression was significantly positive, (b) loneliness could not predict depression through GPIU directly, but (c) loneliness could predict depression through internet gaming disorder to GPIU, (d) loneliness could predict depression through social network use to GPIU, and (e) loneliness could not predict depression through internet gaming disorder to social network use to GPIU. These results provided significant implications for the prevention and reduction of depression in Chinese junior high school students.

## Introduction

Junior high school students are faced with such problems as high academic pressure and monotonous life, which may easily lead to loneliness, depression, and other mental health problems (Elizabeth and John, 2000; Zhang, 2000; Xu, 2012). In recent years, many studies have proved that depression is a mental illness with high prevalence, high chronic recurrence rate, high disease burden, and high suicide mortality (Birk et al., 2019), and there was a high correlation between loneliness and depression (Ren et al., 2020). Studies have demonstrated that adolescents with generalized pathological internet use (GPIU) usually suffer from loneliness (Sukenick, 2012) and depression (Zou et al., 2007). A number of studies have focused on the research of internet gaming disorder and social networking disorder (Bouna-Pyrrou et al., 2015; Wartberg et al., 2020). This study aims at studying the mediating effects of internet gaming disorder, social network use, and GPIU on the association between loneliness and depression.

### Depression

World Health Organization research worldwide shows that depression is affecting physical and mental health in the 21st century (Holden, 2000). From a psychological point of view, depression is the consequence of an ineffective response to life stress, and emotional disorders are the core characteristic, including worthlessness, helplessness and despair, and decreased levels of physical activity (Hammen, 1992). Depression affects emotions as well as thinking, motivation, attention, imagination, behavior, social relationships, and physical conditions, resulting in people feeling lonely and unwell (Jean-Paul et al., 2001; Garcia-Retamero et al., 2015). Junior high school students are in a critical period of physical and mental development and social maturity (Zhang, 2017). The contradiction between ideal and reality often leads to negative emotions, mainly manifested by depression and anxiety, which hinders the healthy development of junior high school students’ mental and physical development (Emamjomeh and Bahrami, 2015). Studies have shown that adolescents’ depressive symptoms increase significantly in frequency and generality (Lewinsohn et al., 1993; Yang et al., 2010; Ferguson, 2020). Many factors affect depression, including both external risk factors and individual susceptibility (Tao, 2006).

### Loneliness and Depression

Clinical and related statistics have demonstrated that loneliness is a common issue of modern people (Huang, 2000; Naama et al., 2019). Adolescence is a particularly vulnerable stage for experiencing feelings of loneliness, which is a significant factor for adolescent health and quality of life (Carvajal-Carrasca and Caro-Castillo, 2009; Danneel et al., 2019). Peplau and Perlman (1979) pointed that loneliness occurred when a person’s social network made him/her less satisfied than he/she expected. Some authors have reported that when social quality declines, the original network of relationships (loss or loss of loved ones, relocation) or lack of social skills (personality factors) could lead to strong loneliness (Vazquez and Garcia, 1997; Teunisse et al., 1999; Tijhuis et al., 1999; Huang, 2000; Van et al., 2017). Hence, loneliness is a subjective feeling of unpleasant suffering caused by social defects (Liu, 1995), and long-term or severe loneliness may trigger certain emotional disorders and reduce mental health (Naama et al., 2019).

Studies found that there was a high correlation between loneliness and depression (Cacioppo et al., 2006; Demir and Kutlu, 2016; Ren et al., 2020). On the one hand, among the negative effects of depression, loneliness is the most common (Ling, 2009). When individuals have negative emotions such as anxiety, pessimism, and disappointment, they tend to experience more feelings of loneliness, loss of help, and desire to be understood. However, on the other hand, loneliness is very detrimental to mental health; a high level of the feeling of loneliness is thought to stimulate depressive symptoms (Wei et al., 2005; Demir and Kutlu, 2016). Qualter et al. (2010) found that the interactive effect of loneliness at 5 and 9 years old predicted depressive symptoms at age 13. Cacioppo et al. (2010) published a recent 5-year longitudinal study that found that loneliness predicted an increase in depressive symptoms at yearly intervals, while depressive symptoms did not predict an increase in loneliness at the same time. A longitudinal study also found a gender-dependent impact of loneliness on depressive symptoms. For females, loneliness could significantly predict the increased depressive symptoms, while for males, loneliness could not (Liu et al., 2019). Therefore, our study will focus on the relationship between loneliness and depression. Based on the above literature discussion, we proposed the following Hypothesis 1: Loneliness could positively predict depression.

### Mediating Roles of GPIU

**FIGURE 1:**
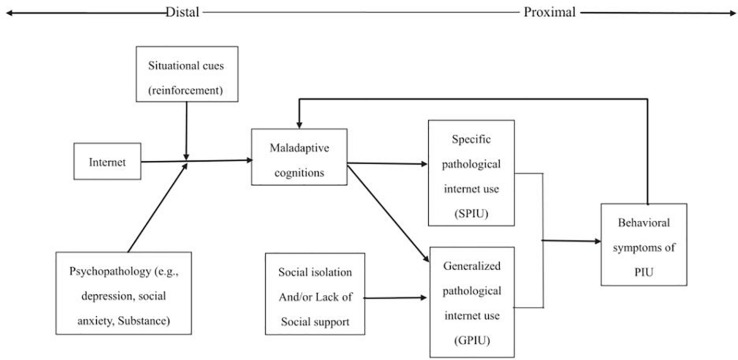
Cognitive behavioral model of pathological internet use (PIU). Adapted from Davis (2001).

Pathological internet use (PIU) refers to a phenomenon of obvious social and psychological damage caused by excessive use of the internet, which means people are unable to control their internet usage (Musetti and Corsano, 2018; Starcevic et al., 2018). Based on the literature, Davis (2001) proposed a cognitive behavioral model of PIU, which highlighted the important role of maladaptive cognition in the development of PIU. The cognitive–behavioral model provided a theoretical explanation for the origin and pathogenesis of GPIU and specific pathological internet use (SPIU) in identifying the etiology of PIU. Davis (2001) proposed two types of PIU (Figure 1): SPIU and GPIU. SPIU is a reliance on the special features of the internet, such as internet gaming disorder and social networks use; GPIU refers to the global set of online behaviors, which contains general, multifaceted internet overuse that perhaps includes no clear destination killing time online (Lopez-Fernandez, 2018). Davis (2001) believed that psychopathology was the distal necessary cause of GPIU/SPIU symptoms. The underlying psychopathology itself did not cause the symptoms of GPIU/SPIU, but it was a necessary factor in its etiology. The key factor in the occurrence of GPIU/SPIU was maladaptive cognition, which was its proximal and sufficient reason.

Generalized pathological internet use, characterized by excessive or compulsive internet use and a preoccupation with and loss of control over this use, results in negative personal, professional consequences (Davis, 2001; Caplan, 2002), and may be detrimental (Greenfield and Yan, 2006). Studies found that adolescents with GPIU usually suffered from loneliness (Young and Rogers, 1998; Engelberg and Sjoberg, 2004; Sukenick, 2012), depression (Jia, 2005; Zou et al., 2007), shyness (Eroglu et al., 2013), poor interpersonal relationships (Sanders et al., 2000; Odaci and Çikrikçi, 2014), cognitive distortion (Lu and Yeo, 2015), and other decreases in well-being (Liu et al., 2012). According to the cognitive behavioral model (Davis, 2001), pre-existing psychosocial problems (depression, or low levels of social support) predispose an individual to GPIU cognitions, behaviors, and negative outcomes. Therefore, investigating the detailed relationships of these variables and GPIU is necessary.

Internet addiction was positively associated with loneliness and depression (Kraut et al., 1998; Young and Rogers, 1998; McKenna and Bargh, 2000; Li et al., 2001; Nie et al., 2002; Young and Kimberly, 2007; Ko et al., 2009). Loneliness is more common among internet addicts (Fan and Yuan, 2018), and loneliness in children and adolescents is one of the predictors of internet addiction (Pontes et al., 2014). Tian et al. (2017) found that the association between loneliness and GPIU was dynamic and bidirectional, and Gao et al. (2018) found that loneliness could predicted GPIU. Then, Demir and Kutlu (2016) noted that loneliness was a significant predictor of internet addiction among college students at ages 17–31. In addition, a study found that teenagers in an internet addiction group had more experiences of online anxiety and depression (Jia, 2005; Zou et al., 2007). Akin and İskender (2011) pointed that if individuals could reduce their internet addiction, they would be able to reduce their depression level. Studies applying longitudinal designs pointed that the GPIU caused depression (Regina et al., 2011), and Park (2009) found that use of the internet tended to increase depression by a longitudinal study. Hence, we will focus on the mediating roles of GPIU between loneliness and depression. So, we proposed Hypothesis 2: Loneliness could predict depression through GPIU.

### Mediating Role of Internet Gaming Disorder and Social Network Use Between Loneliness and GPIU

To improve prevention and intervention of GPIU, its inner working process must be explored, such as an investigation of the intermediary mechanism, which helps to explain how loneliness affects GPIU. The internet gaming disorder and problematic use of social networks belong to SPIU. Young and Kimberly (1998, 2007) conducted a series of studies on the behavioral characteristics of internet addicts. The results suggest that internet addicts tend to prefer online chat and online interactive games. Therefore, Young and Rogers (1998) posited that the internet was not addictive, but special network applications contributed to the occurrence of internet addiction. Gross (2004) research emphasized that the internet was very important for today’s youth and that computer games and the internet communication may lead to social isolation by replacing friends of children or young people. Also, more internet-addicted students indulge in new and exciting online games and two-way interactive online chat (Yao and Yang, 2014; Zhang and Lei, 2015). In recent years, a number of studies have proposed that the concept of internet addiction lacks specificity and focused on the research of internet gaming disorder and social networking disorder (Bouna-Pyrrou et al., 2015; Wartberg et al., 2020).

First, we proposed social network use as a mediator between loneliness and GPIU. Morahan-Martin and Schumacher (2000) found that individuals with high loneliness were more likely to seek social satisfaction on the network to compensate for social deficiencies in real life. Doane (2008) also found individuals’ loneliness experience was an important psychological factor that affected interpersonal communication, and individuals with high loneliness and social anxiety often cannot establish stable interpersonal relationships in real life and often search for alternatives through the network. Błachnio et al. (2016) also found that individuals with high loneliness used mobile social networks more often than individuals with low loneliness. In addition, some studies have asserted that online social networking can reduce more social cues and less direct evaluation by others than offline social networking. People prefer to self-present through social networks, establish and maintain their positive image, and reduce and avoid the level of anxiety that exists in real life (Lee et al., 2014). Thus, teenagers with loneliness are more likely to choose online socializing, which is manifested as pathological social network use.

Another mediator variable is internet gaming disorder. Internet games are a new form of entertainment combining traditional games with the internet, which have strong interactivity and virtuality, and will lead to many behavioral problems among teenagers (Chen and Fu, 2012), such as tired of school, truancy, dropping out of school, and even cybercrime. The emotional deficiency of lonely individuals in real life can be satisfied through social activities and emotional communication through games in the virtual world (Griffiths et al., 2004), which will make lonely people spend more time on online games, and leads to internet gaming disorder. Bozoglan et al. (2013) pointed that loneliness, self-esteem, and life satisfaction were affirmed to account for 38% of the total variance in internet gaming addiction, and loneliness was the most important variable predicting internet addiction. Lee et al. (2019) also proposed the relationship between loneliness and online game addiction. So, we presented Hypothesis 3: Loneliness could predict depression through social network use to GPIU; and Hypothesis 4: Loneliness could predict depression through internet gaming disorder to GPIU.

What is more, at present, more and more people use internet games to socialize online, but there are few relevant studies. Therefore, this study will also explore the chain mediating effect of internet gaming disorder to social network use in loneliness to GPIU. So, we further put forward Hypothesis 5: Loneliness could predict depression through internet gaming disorder to social network use to GPIU.

## Materials and Methods

This study conformed to the code of ethics of the World Medical Association (Declaration of Helsinki) for experiments involving humans and was approved by the Ethics Committee of Shandong Normal University. Additionally, our research obtained written informed consent from the parents of the participants.

### Participants

The participants were from a junior high school in Eastern China, which is an ordinary school and whose students are representative. Students in the first through third grades were included. After eliminating incomplete and repetitive questionnaires, a total of 2211 valid questionnaires were received. Overall, 1087 (49.2%) participants were male and 1124 (50.8%) were female. Their mean age was 13.04 years (aged 10–16 years, *M* = 13.04, *SD* = 1.226).

### Procedures

We obtained informed consent from the school administrators and students before data collection. The paper questionnaire in Chinese was used in this study. To maintain the quality of the investigation, the junior high school students were gathered in a large assembly room to complete their questionnaires with the help of two researchers. The other grades finished the questionnaires with the help of two researchers during one full class period of 45 min.

### Measures

#### UCLA Loneliness Scale

The UCLA Loneliness Scale comprises 20 items, for example, “I am unhappy doing so many things alone.” The items were rated on a four-point scale for frequency (1 = often to 4 = never). The final score was calculated by the total score of all the items, and higher scores indicated greater levels of loneliness. We also conducted CFA, with χ^2^*/df* = 3.846, *RMSEA* = 0.023, and *CFI* = 0.993. Cronbach’s alpha for the UCLA Loneliness Scale was 0.855.

#### Center for Epidemiologic Studies Depression Scale (CES-D)

The CES-D Scale was used to measure the students’ depression, which was adapted to the Chinese language and culture and comprised 20 items, for example, “I felt that I could not shake off the blues even with help from my family or friends.” The students rated each item on a four-point scale from 1 = rarely or none of the time (less than 1 day) to 4 = most or all of the time (5–7 days). To calculate the final CES-D score, the scores of all the items were added. A very high score meant severe depression. We also conducted CFA, with χ^2^*/df* = 3.510, *RMSEA* = 0.022, *CFI* = 0.994, and Cronbach’s alpha was 0.894.

#### Generalized Pathological Internet Use Scale (GPIUS)

To measure the students’ generalized pathological use of the internet, Patricia Gomez’s GPIUS was adapted (Gómez et al., 2017). A total of 11 items were comprised in this version of the GPIUS, for example, “You connected to the internet even though you knew it could get you in trouble.” The items on the GPIUS were rated on a seven-point Likert scale (1 = completely disagree to 7 = completely agree). In the end, the scores of all the items were added to obtain the total GPIUS score. The higher the score, the more serious the GPIU situation was. We also conducted CFA, with χ^2^/*df* = 3.794, *RMSEA* = 0.023, and *CFI* = 0.998. Cronbach’s alpha for the GPIUS was 0.883.

#### Ten-Item Internet Gaming Disorder Test

The Ten-Item Internet Gaming Disorder Test (IGDT-10) comprised 10 items and assesses levels of IGD (Orsolya et al., 2017). In an attempt to operationalize IGD, this instrument used the nine DSM-5 criteria in a brief and simple manner and adopted clear, unambiguous wording for each item. The diagnostic criteria of IGD based on the DSM-5 were strictly followed while considering Petry et al. (2014) recommendations to increase content validity. Each criterion was operationalized using a single item, except for the last criterion, referring to “jeopardy or losing a significant relationship, job, or educational or career opportunity because of participation in internet games” (Orsolya et al., 2017). This criterion was operationalized through two items given its complexity and description of more than one construct. Response options for the 10 items were never, sometimes, and often instead of yes and no. Consequently, the composite score of IGDT-10 was from 0 to 9, and higher scores indicated more severe cases of IGD. The CFA demonstrates that the fitting index is acceptable: χ^2^*/df* = 11.5, *RMSEA* = 0.045, and *CFI* = 0.994. Cronbach’s alpha of the scale was 0.68.

#### Social Network Use Intensity Scale

The original scale used the social strength website questionnaire compiled by Ellison, Steinfield, and Lampe to assess the intensity of youth social networking use (Ellison and Steinfield, 2006). The questionnaire comprises eight items, and the first two items use a self-reporting method to measure the number of individual social networking friends and the average daily spending on social networking sites. The latter six items use the seven-point Likert review method to measure the emotional connection strength of individuals and social networking sites and the extent to which social networking sites integrate individual life. This questionnaire removes the first two items. This measure includes two self-reported assessments of the Chinese network tools QQ and WeChat. Twelve questions measure the intensity of QQ and WeChat use. Six questions are related to WeChat, and QQ is the same. One sample item is as follows: Social networking sites are part of my daily activities. Participants rated each item on a five-point scale, from 1 (I really disagree) to 5 (I very much agree). All scores were standardized and added together, which is the intensity score for social networking sites. The higher the score, the greater the intensity of social networking sites. We also conducted CFA, with χ^2^*/df* = 35.99, *RMSEA* = 0.138, CFI = 0.865, and Cronbach’s alpha was 0.877.

### Statistical Analysis

**FIGURE 2:**
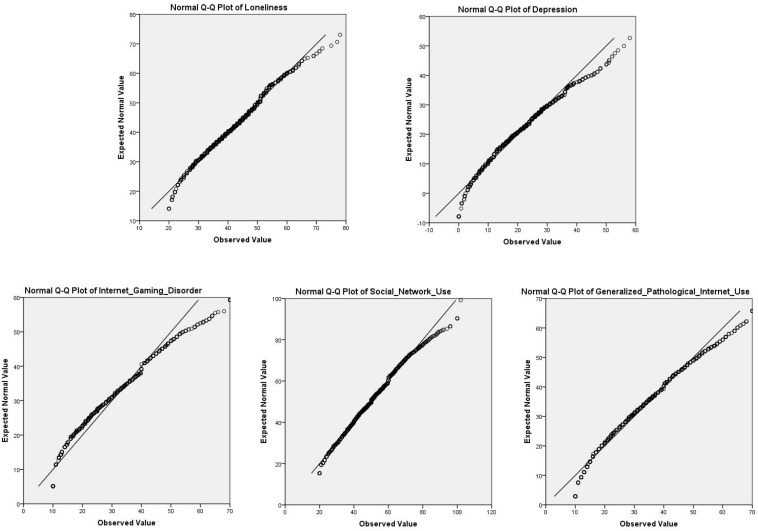
The Q–Q graph of each variable.

Liu pointed that the coefficient test results of normal distribution should be combined with the sample size. In large samples (*n* ≥ 200), the influence of non-normal would be reduced; that is, the assumption of normal distribution in large samples could be slightly violated (Liu, 2019). In this study, the test of the normality of variables was carried out by means of graphic test (Q–Q graph). First of all, as shown in the Figure 2, the Q–Q graph was used to test the normality of each variable, and the variables basically presented a normal distribution. The missing values were replaced by means. To prevent measurement errors caused by common method bias, we used Harman single factor analysis (Podsakoff et al., 2003). Next, descriptive and correlation analyses were used. Subsequently, a structure equation model was used to evaluate a multiple mediation model for the roles of social network use, internet gaming disorder, and GPIU in the relationship between loneliness and depression.

Moreover, parceling strategy was adopted to improve the quality of the model fit and the indicators (Tian et al., 2017). Firstly, we executed a factor analysis. Then, we sorted the items of each observed variable from highest to lowest according to factor loading size (Rogers and Schmitt, 2004). We sequentially assigned the highest and lowest remaining items to each package in turn and alternated the package until all items were completed.

The fits of the model were assessed using the chi-square (χ^2^) test, the RMSEA, the CFI, and the TLI. Considering the χ^2^ test is easily affected by the size of samples, model fit indices were used as the major standard to assess the model fit (Gao et al., 2018). The CFI and TLI ranged from 0 to 1, with values above 0.90 representing sufficient model fit (Hoyle, 1995). A criterion of thumb for the RMSEA is that values ≥0.10 represent poor fit, values between 0.05 and 0.08 represent a reasonable error of approximation, and values ≤0.05 represent close approximation (Cudeck and Browne, 1992).

We used the structural equation model to investigate the relationship of these variables through Mplus 7.0. The bootstrapping method was conducted to test the mediation effects. According to the mediation effect test procedure, the direct effect of loneliness on depression was first tested, and then the significance of the path coefficient and the fitting of the model after the addition of the mediator variables social network use, internet gaming disorder, and GPIU were tested. From 1000 resamples of the data, this method produced 95% bias-corrected confidence intervals of these effects. When the confidence interval did not contain zero, it meant that there was a significant effect at *p* < 0.05.

We conducted the common variance analysis to measure whether common method biases existed in this study. The χ^2^ test of Bartlett’s test of sphericity was significant. Then, we extracted 12 eigenvalues greater than 1 after a principal component analysis. The first factor to explain the variance was 19.305%. The results were less than the critical standard of 40% (Podsakoff et al., 2003), indicating that these instruments had no problem with the common method biases.

## Results

### Descriptive Statistics and Correlation Analysis

Table 1 shows the descriptive statistics and Pearson correlation of social network use, loneliness, internet gaming disorder, depression, and GPIU. The results indicated that loneliness had a weak negative correlation with social network use, and there was a significant positive correlation between other variables.

**TABLE 1:** Descriptive statistics and correlation matrix of all variables.

Variable	*M*	*SD*	1	2	3	4	5
Loneliness	41.42	9.16	1				
Social network use	49.83	14.23	−0.046*	1			
internet gaming disorder	24.76	12.99	0.199**	0.344**	1		
GPIU	31.02	12.73	0.221**	0.305**	0.616**	1	
Depression	17.34	10.14	0.626**	0.103**	0.318**	0.341**	1

*N* = 2211. * *p* < 0.05, ** *p* < 0.01.

### Testing for Multiple Mediating Roles of Social Network Use, Internet Gaming Disorder, and GPIU

**FIGURE 3:**
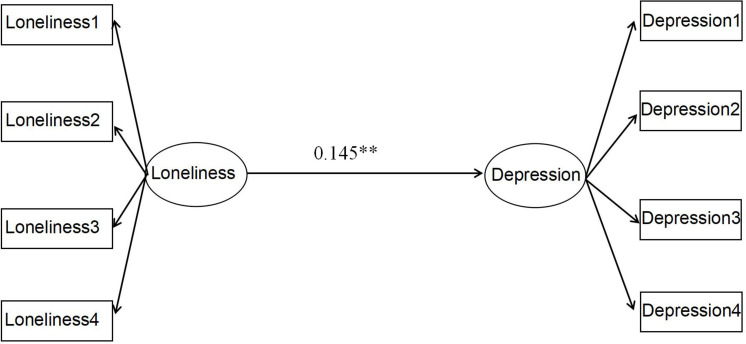
Total effect model. Path values are the path coefficients. ^∗∗^*p* < 0.01. The latent variables loneliness and depression were divided into four packages through the packaging strategy.

First, the finding demonstrated that the path coefficient of loneliness to depression was significantly positive (Figure 3). The fits of this model were χ^2^*/df* = 5.78, *RMSEA* = 0.051, *NFI* = 0.978, *CFI* = 0.975, and *TLI* = 0.968, which indicated that the model was appropriate and acceptable.

**FIGURE 4:**
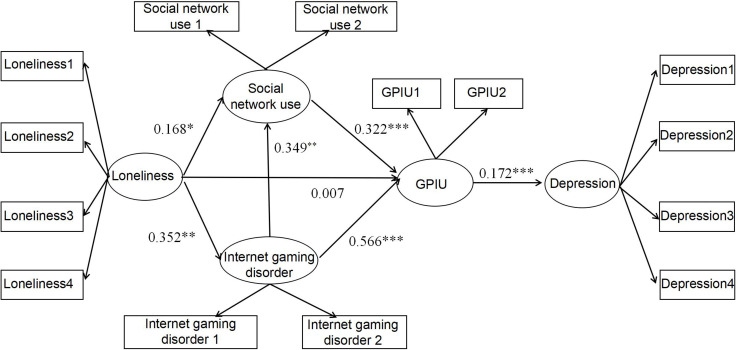
Multiple mediation model. ^∗^*p* < 0.05, ^∗∗^*p* < 0.01, ^∗∗∗^*p* < 0.001. The latent variables loneliness and depression were divided into four packages through the packaging strategy, and the latent variables social network use, internet gaming disorder, and GPIU were divided into two packages through the packaging strategy.

Second, the indirect effects of internet gaming disorder, social network use, and GPIU between loneliness and depression were explored (Figure 4). The path coefficients of loneliness to internet gaming disorder/social network use, internet gaming disorder to social network use, internet gaming disorder/social network use to GPIU, and GPIU to depression were significantly positive. Specifically, the direct path coefficients of loneliness to GPIU were not significant.

Third, most of the mediated effects paths were significant (Table 2). Specifically, loneliness could predict depression through social network/internet gaming disorder to GPIU. However, loneliness could not predict depression through GPIU. The direct effect of loneliness on depression was significant, but path loneliness to GPIU was not significant after the addition of two mediator variables. What is more, loneliness could not predict depression through internet gaming disorder to social network use to GPIU.

**TABLE 2:** Test of the total effect model and mediation effect model.

Path	Standardized path coefficient	95% confidence interval	
		Lower	Upper
**a. Total effect model**			
Loneliness → depression	0.044**	0.012	0.078
**b. Multiple mediation model**			
Loneliness → GPIU → depression	0.001	–0.020	0.023
Loneliness → internet gaming disorder → GPIU → depression	0.034**	0.015	0.054
Loneliness → social network use → GPIU → depression	0.009*	0.002	0.017
Loneliness → internet game disorder → social network use → GPIU → depression	0.005	–0.003	0.011

*N* = 2211. *p < 0.05, **p < 0.01.

## Discussion

The purpose of this study was to explore the relationship between loneliness and depression and the mediating effects of social network use, internet gaming disorder, and GPIU, in order to further explore the underlying mechanisms by which loneliness affects depression. The total effect model indicated that loneliness could positively predict depression, and Hypothesis 1 was supported. The multiple mediation model showed that loneliness did not affect depression through GPIU, which did not support Hypothesis 2. The model also indicated that the two mediators of internet gaming disorder and social network use parallel mediated the relationship between loneliness and depression through GPIU, which supported Hypotheses 3 and 4. However, the two mediator variables could not have a sequential relationship between loneliness and depression, which did not support Hypothesis 5.

### Loneliness and Depression

The result showed that the path coefficients of loneliness to depression were significantly positive, which was consistent with previous studies (Wei et al., 2005; Wang et al., 2008; Cacioppo et al., 2010; Demir and Kutlu, 2016). Studies have shown that loneliness is still significantly associated with depressive symptoms after controlling for demographic information (including gender, age, financial income, marital status, etc.) and risk factors that jointly influence loneliness and depression (including hostility, social support, stressors, etc.) (Cacioppo et al., 2006; Demir and Kutlu, 2016; Ren et al., 2020). Loneliness is associated with personality disorders, psychiatric disorders, and suicide, which can also impair executive control and increase depressive symptoms (Richman and Sokolove, 1992; Neeleman and Power, 1994; Deniro and Dorothy, 1995; Cacioppo et al., 2006; Mushtaq et al., 2014).

Several reasons have been proposed to explain why loneliness is connected with the development of depression. First, lonely individuals always have negative perceptions of things, are susceptible to negative emotions, and often show hostility, and hostility is significantly related to depression (Doering et al., 2009; Li Y. L. et al., 2019). Second, students affected by short-term loneliness and long-term loneliness are more likely to be depressed and unwilling to communicate with others. If these students do not find a proper channel for catharsis, this situation may not improve and may be exacerbated (Van et al., 2017; Sun and Liu, 2018). In addition, De Jong-Giervald (1987) defined loneliness as a subjective social isolation, accompanied by personal perceptions, unacceptable painful experiences resulting from isolation or lack of contact with others, and long-term loneliness that has an adverse effect on the health of lonely patients and can cause emotional disorders such as depression. Long-term loneliness can have a negative impact on their blood pressure and immunity, leading to poor health and emotional disorders such as depression (Dai, 2017; Naama et al., 2019).

However, there is a co-existence between depression and loneliness (Cacioppo et al., 2006; Demir and Kutlu, 2016; Ren et al., 2020); people with depression are more likely to feel lonely due to actively avoiding people (Xue, 2017). What is more, individuals with depression tend to lack confidence and have low self-evaluation, which may lead to feelings of loneliness (Guo et al., 2016). Therefore, further longitudinal studies are needed to determine the causal relationship between loneliness and depression.

### The Key Mediating Role of GPIU

Loneliness, internet gaming disorder, and social network use were significantly associated with GPIU, and GPIU was significantly related to depression. The results of this study supported the theory by Kraut et al. (1998), which pointed that when researchers controlled possible mediating variables, loneliness, depression, and daily stress were positively correlated with greater internet usage. The important finding was that further use of the internet was related to an increase in depression over a subsequent period of time (Kraut et al., 1998), which was found in more later studies (Jia, 2005; Zou et al., 2007; Park, 2009; Akin and İskender, 2011; Regina et al., 2011). Several studies have mentioned the theoretical basis for the link between loneliness, depression, and internet addiction (Davis, 2001; Chung, 2013).

This study found that loneliness was not directly related to depression through GPIU, which was not consistent with previous assumptions. Previous studies supported the idea that some people used the internet to cope with negative emotions such as sadness, anxiety, or loneliness (Scherer, 1997; Muñoz-Rivas et al., 2010; Zhou et al., 2017) or to escape psychological problems (Morahan-Martin and Schumacher, 2000). Affected by Davis (2001) cognitive behavioral model, Caplan (2002) argued that people with psychological problems were more likely to choose to communicate online than face to face in order to compensate for their social skills. However, if people use the internet excessively or inappropriately, it will have a bad effect on body and mind (Li, 2020).

The possible reason about this study result is that loneliness could actually affect depression through GPIU, but as a result of SPIU (internet gaming disorder and pathological social network use) as a mediation variable in the model, the direct effect of loneliness on GPIU is weakened. According to Davis’ cognitive behavior model of PIU (Davis, 2001), SPIU and GPIU have a side-by-side relationship, but based on the literature (Bouna-Pyrrou et al., 2015; Wartberg et al., 2020), we speculate that psychopathology and maladaptive cognition could directly lead to SPIU (e.g., internet gaming disorder and pathological social network use) in some cases and then lead to GPIU. The key to the occurrence of PIU is the emergence of social network use and internet gaming disorder, which is a sufficient cause of PIU symptoms, and which ultimately leads to the development of PIU symptoms, such as depression. Previous studies revealed that pathologic internet use could be associated with depression, substance-related disorders, obsessive–compulsive symptoms, low self-esteem, and attention deficits (Shapira et al., 2000; Tsai and Lin, 2003; Kim et al., 2006). What is more, Liang et al. (2016) pointed that in female adolescents, internet addiction was found to significantly predict subsequent depression, indicating that internet addiction leads to depression and supporting the social displacement hypothesis. The possible reason is that teenagers with internet addiction are addicted to the virtual world, which interferes with their interpersonal relationship in the real world, resulting in the lack of face-to-face communication with others and lack of social support in the real world, so they are prone to become depressed (Kraut et al., 1998; Yang, 2016).

### Mediating Roles of Social Network Use and Internet Gaming Disorder

In the mediation process of loneliness to internet gaming disorder/social network use to GPIU to depression, the results of this research were consistent with other related research (Morahan-Martin and Schumacher, 2000; Yan, 2009). A possible reason for this similarity is that loneliness is a factor that has been frequently associated with GPIU (Morahan-Martin and Schumacher, 2000; Davis, 2001; Caplan, 2007; Ceyhan and Ceyhan, 2008; Kim et al., 2009; Dowling and Brown, 2010; Odaci and Kalkan, 2010; Ang et al., 2012; Barthakur and Sharma, 2012). Additionally, social network use and internet gaming disorder are two important forms of internet addiction, and people maintain social connections through these two forms. Loneliness occurs when social connections are cut off (Blazer, 1983; Lv, 2016), and the emergence of loneliness makes people try to reconnect or establish new connections (Weiss and Bowlby, 1975; Cagan, 2009). Social networking chat and internet gaming are critical ways for interpersonal interactions and relaxation (Li, 2015). It is worth noting that it is wise to distinguish between GPIU and specific network-related behavior in question. Research on specific behaviors that individuals engage in on the internet shows that individuals are not addicted to the internet media itself but to the specific behaviors or content they engage in or access (Starcevic, 2013; Brand et al., 2016).

Social networks are an emerging network of communication media used primarily to maintain existing relationships (Antheunis et al., 2010; Boyd and Ellison, 2010). Notably, supplementations to offline interpersonal communication are important (Kujath, 2011). Behavioral experiments have also shown that social site status updates have become an important aspect of social networking sites, and lonely people can significantly reduce the loneliness of personal experiences through social networking sites (Deters and Mehl, 2013). Positive online feedback may be one of the reasons why social network use reduces individual loneliness. Social networks based on acquaintances make it easier for individuals to obtain supportive feedback from, for example, friends and classmates. From a theoretical point of view, Social compensation model theory believes that individuals with less social connections may use the internet to obtain compensation for interpersonal interactions, to meet the psychological needs that are lacking in real life, and to obtain satisfactory social relationships (Kraut et al., 1998). In addition, Suler (1999) found that the need for social interaction is one of the needs of internet addiction. When the satisfaction brought by the internet to the lonely individual is stronger than the satisfaction in real life, the lonely individual may be addicted to the internet, which is consistent with the satisfaction theory. However, if the lonely individual relies too much on network compensation and without restraint, it may become a PIU (Zhang et al., 2017). What is more, the model of the poor becoming rich is derived from the theory of social compensation, which believes that the network can enhance the connection between individuals and others and promote the formation of intimate relationships (Tan, 2015). The social lack of lonely individuals in real life can be satisfied through the interactive nature of online games and supportive interpersonal communication (Valkenburg et al., 2005).

What is more, the concealment of the network and the irritability of internet games greatly satisfy the self-demand that they cannot achieve in reality. Internet games have also become a passive lazy escape response to problems. According to the reinforcement theory (Villere and Hartman, 1991), the increasing use and reinforcement of technology are the elements of internet gaming disorder. The improvement of game technology will make individuals psychologically satisfied and thereby escape and eliminate the discomfort brought to them by real life sense. Through continuous learning and upgrading skills in the game, teenagers show their extraordinary wisdom through superb technology, win the attention of all players, and enjoy the sense of accomplishment brought by self-realization (Kuang, 2017). Due to lack of social support, lonely individuals are more likely to produce negative self-awareness, such as self-denial and inferiority (Hou, 2013). The improvement of technology in online games will help lonely people to generate positive self-evaluation and achieve a sense of accomplishment, which in turn increases their dependence on online games, leading to addictive behaviors (Bozoglan et al., 2013; Lee et al., 2019).

### Implications for Prevention of Depression

The results of this study were of great significance to depression prevention and intervention strategies for Chinese students. First of all, junior high school students under great study pressure and housing pressure were very prone to depression. Therefore, depressed students deserve more attention from teachers and parents. Teachers and school doctors are required to improve their quality, pay attention to the psychological status of students, and treat students with a positive attitude. Parents should also strive to provide good parenting for their children. Through appropriate expectations and a harmonious family environment, parents can provide children a benign stimulus so that they can adopt a positive coping style to apply to various contradictions and prevent the formation of adverse emotions (Zhu et al., 2003; Li T. H. et al., 2019). Furthermore, two strategies can help students cope with depression. One strategy is to overcome the behavioral symptoms of internet addiction, starting with reducing the use of social networking and internet gaming disorder (SPIU). In this regard, the cognitive behavior model is useful (Davis, 2001). Moreover, other interventions could focus on initial variables such as loneliness because loneliness is very unfavorable to mental health and promotes depression (Wei et al., 2005). Thus, regular group counseling activities should be organized for junior high school students to promote interaction among students and reduce loneliness, SPIU, GPIU, and depression, respectively.

### Limitations and Future Directions

Although this study was conducive to understanding the relationship between loneliness and depression in Chinese students, there were still some limitations. First, this study was a cross-sectional study, and its results could not provide a causal relationship. Therefore, further research could explore the causal relationship between these variables through experiments or longitudinal studies, such as loneliness and depression. Another limitation was that the questionnaire survey used in this study may cause some errors. For example, self-reported questionnaires were influenced by social desirability. Hence, further research may apply more professional surveys (e.g., Survey Monkey or Google Forums) to directly record time spent, eliminating coding data errors and controlling the answers at random. In addition, the convenience sampling may limit the generalizability of the result, so if there is an opportunity, we will expand the sample and increase the randomization level of the sample. What is more, other mediators of the relationship between loneliness and depression (such as meditation), as well as how social network use and internet gaming disorders predict depression, and how the two-way relationship needs to be explored. Last but not least, this study was conducted in the context of Chinese culture, so the cross-cultural applicability of the conclusions must be further verified.

## Conclusion

In this study, we discussed the mediating effects of internet gaming disorder, social network use, and GPIU on loneliness and depression, and the conclusions were as follows: (a) loneliness could positively predict depression; (b) loneliness could not predict depression through GPIU directly; (c) loneliness could predict depression through internet gaming disorder to GPIU; (d) loneliness could predict depression through social network use to GPIU; and (e) loneliness could not predict depression through internet gaming disorder to social network use to GPIU.

## Data Availability Statement

The datasets for this manuscript are not publicly available because the datasets are used only for the team of this article by the permission of the guardians. Requests to access the datasets should be directed to PW, 122394108@qq.com.

## Ethics Statement

The studies involving human participants were reviewed and approved by the Ethics Committee of Shandong Normal University. The guardians of the participants provided written informed consent to let them participate in this study.

## Author Contributions

PW was the research designer. JW and YY were in charge of writing. XZ and YT participated in the discussion and offered suggestions. YS was the corresponding author. All authors contributed to the article and approved the submitted version.

## Conflict of Interest

The authors declare that the research was conducted in the absence of any commercial or financial relationships that could be construed as a potential conflict of interest.
